# What Goes “99-Thump?”

**DOI:** 10.1371/journal.pbio.1002006

**Published:** 2014-11-25

**Authors:** Roland G. Roberts

**Affiliations:** Public Library of Science, Cambridge, United Kingdom

A centipede with a wooden leg, of course. Laying aside for the moment the issue of whether any centipede does have 100 legs, this childish joke is an indicator of the special place that these creepy-crawliest of arthropods occupy in our collective psyche. Here's another one—a 150-year-old poem called the Centipede's Dilemma, usually attributed to Katherine Craster: “A centipede was happy—quite!/Until a toad in fun/Said, ‘Pray, which leg moves after which?’/This raised her doubts to such a pitch,/She fell exhausted in the ditch/Not knowing how to run.” This poem in turn gave its name to the Centipede Syndrome—the phenomenon whereby concentrating on a normally unconscious task compromises our ability to perform it.

The arthropods are one of Earth's real success stories—there are more species of arthropod than in any other animal phylum, but our knowledge of the genomic basis of arthropod biology is massively skewed towards insects. The phylum Arthropoda is conventionally subdivided into five subphyla, four of which are still with us. The hexapods (insects and their kin) scored the first arthropod genome sequence—that of the lab workhorse *Drosophila melanogaster*, published back in 2000—followed by many other insect genomes since. The chelicerates (spiders, scorpions, horseshoe crabs, etc.) and crustaceans (shrimps, crabs, barnacles, etc.) had to wait 11 more years for the publication of the sequences of *Tetranychus urticae* (red spider mite) and *Daphnia pulex* (water flea), respectively. Trilobites haven't been seen for a while now, so this just leaves the myriapods as the only extant arthropod subphylum with no sequenced genome.

The myriapods comprise two major classes—the largely vegetarian millipedes and the rather viciously carnivorous centipedes—plus two relatively insignificant ones (the tiny, stubby pauropods and the translucent symphylans). Myriapods arose more than 400 million years ago (and possibly as far back as the Cambrian), probably as an independent land invasion from the insects (they're all terrestrial). The most obvious shared trait of the subphylum is the large number of near-identical segments, most bearing one or two pairs of legs. There are now at least 13,000 recognized myriapod species across the world; about a quarter of these are centipedes: predators with 30–400 legs, venom-injecting pincers on their heads, simple eyes, and a predilection for skulking.

As well as the intriguing biology of the myriapods themselves, there are other reasons to be interested in their genomes. The myriapods are the presumed sister-group of the crustaceans and insects—creatures of enormous importance to us, whether as food, pests, pollinators, disease vectors, or biological models. This means that myriapod genomes could present a unique opportunity to reconstruct the common ancestor of this group of subphyla, which together form the “mandibulates.”

**Figure 1:**
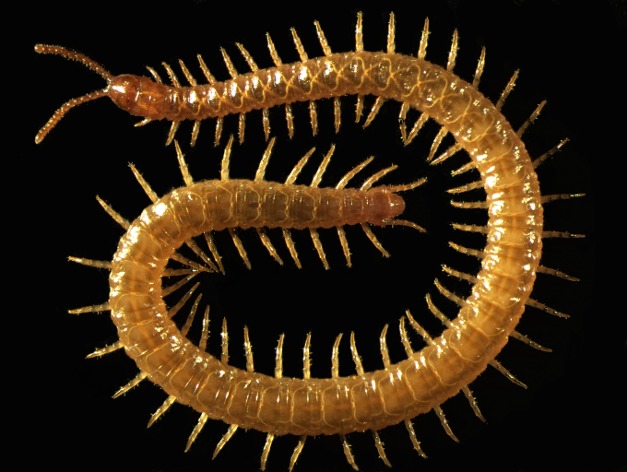
*Strigamia maritima*, whose genome sequence has just been revealed.

An international consortium of researchers from 52 different institutions now publish in *PLOS Biology* the sequence of the genome of *Strigamia maritima*, a Northern European beach bum centipede that is easily collected in the wild, has been the model myriapod for developmental studies, and has a conveniently modest-sized genome (Figure 1). There are some downsides to the choice of this species, in that *S. maritima* is atypical in not having eyes and springing from its egg with the full adult complement of segments, but otherwise it seems a fine choice for our first glimpse of a myriapod genome.

At an estimated 290 megabases, the genome is about one-tenth the size of ours, and the authors describe about two-thirds of this, the remaining third being too highly repetitive. They also re-sequenced four individuals to capture some of the genetic variation that's present in the wild. The authors were able to identify about 15,000 genes (we have more than 20,000), distributed across one pair of very large chromosomes and seven pairs of tiny ones, including X and Y sex chromosomes.

The arrangement of the genes is relatively conservative, resembling that of genomes from other animal phyla, and much less scrambled than are the genomes of some model arthropods such as flies. Together with some of the more conservative insect genomes (silk moth, red flour beetle) and that of the crustacean water flea, this will help fill in our picture of the ancestral mandibulate from which this massive diversity sprung.

Having laid out the basic properties of the genome, the authors take us on a whistle-stop tour of interesting links between the gene complement of the centipede's genome and features of its biology. These include the structure of the Hox gene cluster (a major orchestrator of body plans in other animals), the elements of the centipede's immune system, endocrine system, and major developmental pathways, and a hint that some genes are actively modified by methylation, presumably to regulate them. Many of these are just variations on a theme, but I'll focus on a few nuggets that speak more directly to the evolutionary history of *S. maritima*.

One sense that needs to develop substantially when an animal lineage takes the first steps out of water and onto dry land is the sense of smell. Vertebrates, insects, and myriapods have made this career choice independently, and it's already known that the first two groups of animals have reached different solutions to the problem of detecting chemicals in air. The current study now shows that despite belonging to the same phylum as insects, myriapods have gone down a third route, olfaction-wise. Instead of having the large repertoire of odorant receptors and odorant-binding proteins that insect genomes have, the centipede genome contains expanded sets of two other protein families—gustatory receptors and ionotropic receptors. The authors infer that when the great granddaddy of all myriapods needed to sniff the Cambrian air, it's these protein families that stepped up to provide the instrumentation.

The paper reveals further evidence of the intriguing tendency of evolution to have more than one way to skin a cat—in insects, a single gene called *Dscam* uses massively alternative splicing to generate a dizzyingly diverse array of cell-surface proteins (approximately 150,000) that underlie the complexity of the nervous and immune systems. The centipede appears to achieve the same end, but by using up to a hundred *Dscam*-like genes, each with the potential to undergo a more modest degree of alternative splicing.

*S. maritima* is a member of the Geophilimorph order of centipedes, all of which are blind underground-dwellers. Correspondingly, the authors find no evidence in its genome for any recognised way of sensing light—no obvious photoreceptors, and no components of the circadian clock. To check whether this is a general feature of centipedes or merely a characteristic of this subterranean group, the authors check transcripts from the house centipede *Scutigera coleoptrata*. This animal has genes encoding proteins needed for light detection and circadian behaviour, suggesting that this wholesale loss is a geophilimorph-specific sacrifice, dispensing with genes that have no role to play in the underworld.

The description of the *S. maritima* genome surely heralds a new era in myriapod biology, but in the interim some of you will still be wondering whether any centipede lives up to the eponymous leg number. The answer, barring accidents or prostheses, is apparently no, as all centipedes have odd numbers of leg-bearing segments. Gratifyingly, however, *S. maritima* comes very close—although their segment numbers vary somewhat, I count 94 legs on the animal pictured here, and animals with 98 legs are common.

**Chipman AD, Ferrier DEK, Brena C, Qu J, Hughes DST, et al. (2014) The First Myriapod Genome Sequence Reveals Conservative Arthropod Gene Content and Genome Organisation in the Centipede** ***Strigamia maritima*** **.** doi:10.1371/journal.pbio.1002005

